# A novel dual MEK/PDK1 inhibitor 9za retards the cell cycle at G_0_/G_1_ phase and induces mitochondrial apoptosis in non-small cell lung cancer cells

**DOI:** 10.7717/peerj.9981

**Published:** 2020-10-02

**Authors:** Rangru Liu, Zutao Yu, Zhuo Chen, Danqi Liu, Fengying Huang, Qianbin Li, Gaoyun Hu, Xinan Yi, Xi Li, Honghao Zhou, Zhaoqian Liu

**Affiliations:** 1 Key Laboratory of Tropical Translational Medicine of the Ministry of Education & Hainan Key Laboratory for Research and Development of Tropical Herbs, School of Pharmacy, Hainan Medical University, Haikou, People’s Republic of China; 2 Department of Clinical Pharmacology, Xiangya Hospital, Central South University, Changsha, People’s Republic of China; 3 Hunan Key Laboratory of Pharmacogenetics, National Clinical Research Center of Geriatric Disorders, Xiangya Hospital, Central South University, Changsha, People’s Republic of China; 4 Department of Medicinal Chemistry, Xiangya School of Pharmaceutical Sciences, Central South University, Changsha, People’s Republic of China; 5 Department of Pharmacy, Xiangya Hospital, Central South University, Changsha, People’s Republic of China; 6 Key Laboratory of Tropical Diseases and Translational Medicine of the Ministry of Education & Hainan Provincial Key Laboratory of Tropical Medicine, Hainan Medical University, Haikou, People’s Republic of China; 7 The United Laboratory for Neurosciences of Hainan Medical University and the Fourth Military Medical University, Haikou, People’s Republic of China

**Keywords:** A dual MEK/PDK1 inhibitor, 9za, Cytotoxicity, Cell cycle arrest, Mitochondrial apoptosis, Non-small cell lung cancer

## Abstract

**Background:**

A novel dual MEK/PDK1 inhibitor named 9za has been synthesized by our research team. Preliminary study showed that 9za possessed potent cytotoxicity and proapoptosis in non-small cell lung cancer (NSCLC) cells. Nevertheless, the precise underlying mechanism is vague.

**Methods:**

In this work, we adopted the MTT assay, the Cell Cycle Detection Kit, and the JC-1 staining assay to detect the cell viability, the cell cycle distribution and the mitochondrial membrane potential (MMP), respectively. Cell apoptosis was measured by the morphology observation under a light microscope, Annexin V-FITC/propidium iodide (PI) apoptosis detection and the colorimetric TUNEL assay. Western blot was used to monitor the cell cycle-, apoptosis-related proteins and relevant proteins involved in the signaling pathways.

**Results:**

The MTT assay demonstrated that 9za sharply decreased the viability of NSCLC cells. Cell cycle analysis revealed that low concentrations of 9za arrested the cell cycle at the G_0_/G_1_ phase , which was further confirmed by the decreased levels of Cyclin D1, cyclin-dependent kinase 4 (CDK4) and cyclin-dependent kinase 6 (CDK6). Additionally, morphological observations, Annexin V-FITC/propidium iodide (PI) apoptosis analysis and TUNEL assays indicated that high concentrations of 9za induced cell apoptosis. Furthermore, the JC-1 staining assay revealed that the mitochondrial membrane potential was downregulated following 9za exposure. Western blot also showed that 9za markedly decreased the expression levels of total Bcl-2, Cytochrome C in the mitochondria and BCL2 associated X (BAX) in the cytoplasm. However, the levels of BAX in the mitochondria, Cytochrome C in the cytoplasm, active caspase-9, active caspase-3 and cleaved–PARP showed the opposite changes. Moreover, the dose-dependent decreased phosphorylation levels of PDK1, protein kinase B (Akt), MEK and extracellular signal regulated kinase 1/2 (ERK1/2) after 9za treatment verified that 9za was indeed a dual MEK/PDK1 inhibitor, as we expected. Compared with a single MEK inhibitor PD0325901 or a single PDK1 inhibitor BX517, the dual MEK/PDK1 inhibitor 9za could strengthen the cytotoxic and proapoptotic effect, indicating that the double blocking of the MEK and PDK1 signaling pathways plays stronger cell growth inhibition and apoptosis induction roles than the single blocking of the MEK or PDK1 signaling pathway in NSCLC cells. Our work elucidated the molecular mechanisms for 9za as a novel drug candidate against NSCLC.

## Introduction

Lung carcinoma is a leading tumor type with ranking first and second for the estimated new cancer cases and cancer-related deaths, respectively (Siegel, Miller & Jemal, 2018). Approximately 85% of pulmonary carcinomas are non-small cell lung cells (NSCLC), which is regarded as a particularly aggressive and deadly subtype due to its long latency, rapid progression and intrinsic or acquired drug resistance. Although targeted therapy such as immune checkpoint inhibitors including anti-programmed death-1 (PD-1) and programmed death-ligand 1 (PD-L1) have recently improved the therapeutic efficacy for certain NSCLC patients (Borghaei et al., 2015; Brahmer et al., 2015), the aggressive characteristics of NSCLC have made it an intractable issue. Most patients have not achieved satisfactory curative efficacy due to drug resistance, poor selectivity of chemotherapeutics and high recurrence rates, highlighting the urgency to exploit novel types of chemotherapeutic drugs for lung cancer.

**Figure 1:**
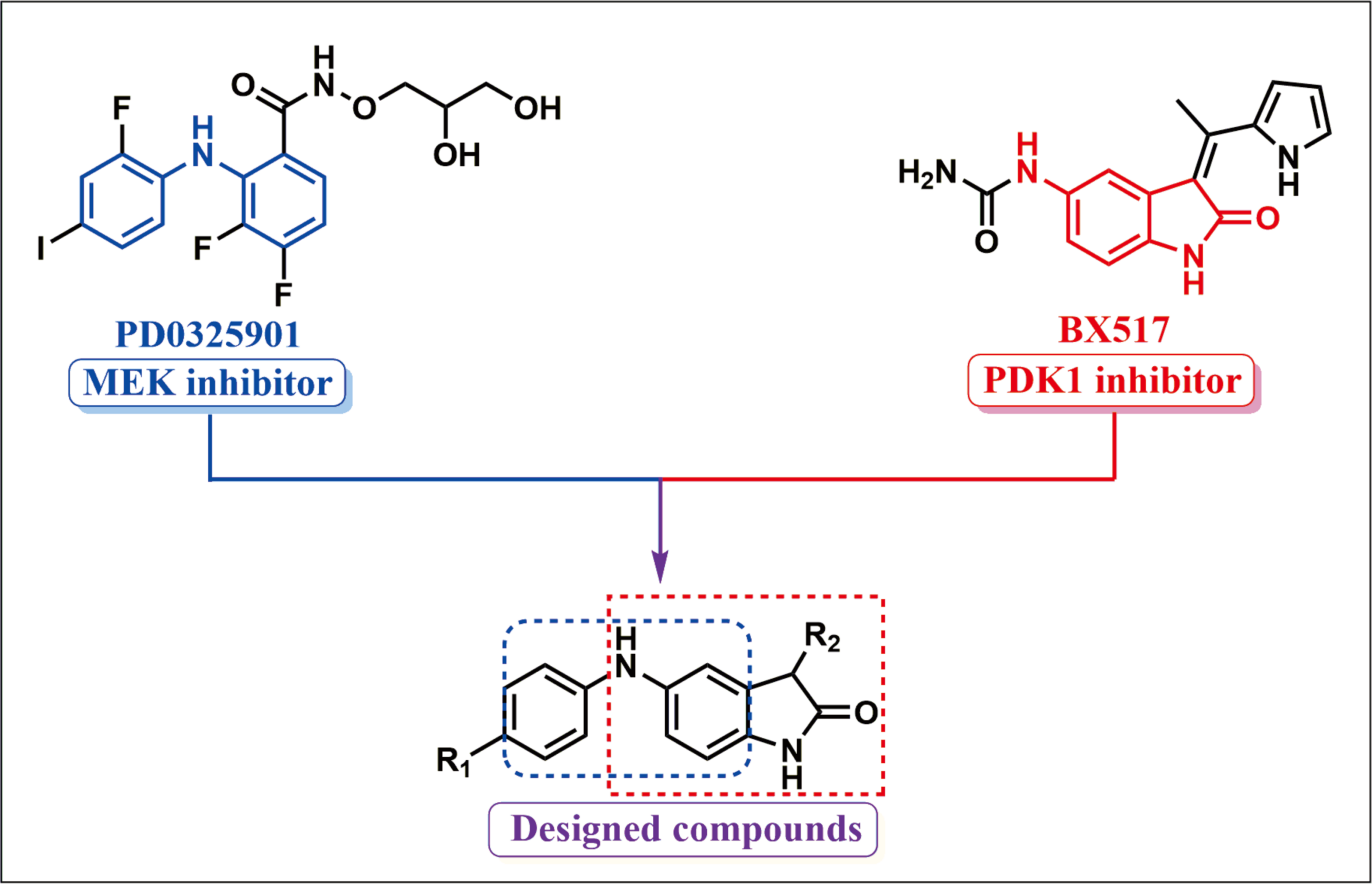
Synthetic design concept of novel dual MEK/PDK1 inhibitors.

**Figure 2:**
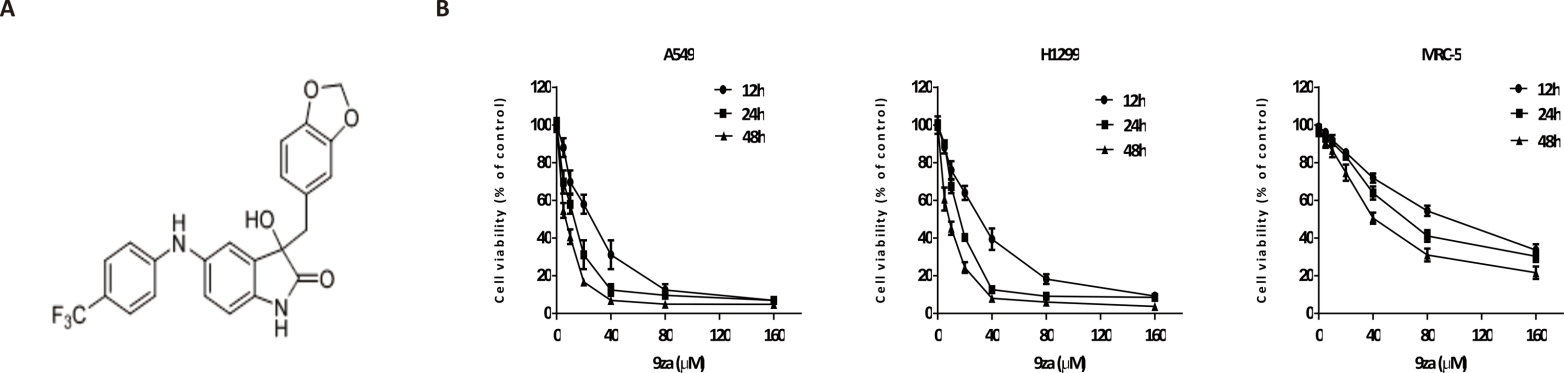
9za has potent cytotoxic effects in NSCLC cells. (A) The chemical structure of 9za. (B) Cell viability was conducted by the MTT assay in A549, H1299 and MRC-5 cells, which were treated with 9za at 0, 5, 10, 20, 40, 80, 160 µM for 12, 24, 48 h, respectively (*n* = 6).

The RAF/MEK/ ERK and PI3K/AKT/mTOR signal pathways control fundamental physiological processes including cell differentiation, metabolism, proliferation, cell death and survival. Constitutive activation of the signal pathways is a required hallmark of cancer and has been implicated in the initiation, progression and metastasis in lung cancer. Growing evidence has suggested that the dual blocking of the RAF/MEK/ERK and PI3K/PDK1/Akt signal axes is an effective means to exploit novel drug candidates for cancers (Ciuffreda et al., 2014; Temraz, Mukherji & Shamseddine, 2015). To acquire a novel type of drug candidate with high anti-tumor potency for lung cancer, guided by the fragment-based drug design (FBDD), a string of new double MEK/PDK1 inhibitors was discovered through merging the functional structures of PD0325901 (a MEK inhibitor) and BX517 (a PDK1 inhibitor) (Fig. 1). Hence, a library of 43 new compounds was established and screened for anti-cancer efficacy. As a result, we found that these new compounds had anti-cancer activity with different degree. The specific synthetic routes and IC_50_ in A549 and H460 cells of the new compounds were summarized in Yu’s (2019) study. Notably, compound 9za (the chemical structure is shown in Fig. 2A) showed the most potent anti-cancer activity among 43 new compounds in NSCLC cells so that it was selected for further research. Our previous study showed that 9za promoted autophagy via the PDK1/Akt/mTOR signal pathway and that autophagy played a pivotal role in 9za-induced cytotoxicity and pro-apoptosis in NSCLC cells (Liu et al., 2019b). However, the exact underlying mechanism of pro-apoptosis for 9za is unclear until now.

Presently, most chemotherapeutic drugs achieve anti-cancer activity through inducing tumor cell apoptosis, which is a complex type of programmed cell death (Qi et al., 2018; Zhang et al., 2018). Apoptosis is highly controlled and regulated by multiple signaling pathways (Fu et al., 2016). Apoptosis might be initiated by two major pathways: the extrinsic and intrinsic (i.e., mitochondrial pathway) cell death pathways (Fan et al., 2016; Huang et al., 2015). Although extrinsic and intrinsic apoptosis have unique initiating steps, both pathways induce cell apoptosis by activating caspase cascades.

Since 9za is designed as a new dual MEK/PDK1 inhibitor, does 9za block both the RAF/MEK/ ERK and PI3K/AKT/mTOR signal pathways? Is 9za a good drug candidate for NSCLC? In this study, we showed that 9za markedly inhibited the cell proliferation of NSCLC cells. Moreover, low and high concentrations of 9za could induce cell cycle arrest and mitochondrial apoptosis, respectively. Further studies revealed that 9za induced apoptosis through the PDK1/Akt and MEK/ERK signaling axes. Therefore, our findings elucidated the mechanism of action of 9za against NSCLC cells and provide an experimental basis to develop 9za as a novel type of drug candidate against NSCLC.

## Materials & Methods

### Cell culture

All of the cell lines were bought from Shanghai Cell Bank of the Chinese Academy of Sciences. Two human NSCLC cells (A549 and H1299) cultured in Dulbecco’s modified Eagle’s medium (DMEM) and one human healthy lung fibroblast cell line (MRC-5) maintained in Minimum Essential Medium (MEM) were cultured in an incubator with 5% CO_2_ at 37 °C. Both cell culture media were supplemented with 10% fetal bovine serum (Gibco, Grand Island, USA) and 10 U/ml of penicillin-streptomycin. Both the cell treatment time of 9za and cell seeding incubation time were 24 h unless otherwise specified.

### Reagents and antibodies

Compound 9za (HPLC purity: 96.9%) was synthesized by Central South University. The Annexin V-FITC/PI Apoptosis Kit, Cell Cycle Detection Kit, Colorimetric TUNEL System, Mitochondrial Membrane Potential Assay Kit with JC-1 and MTT were purchased from MultiSciences (Hangzhou, China), KeyGEN (Nanjing, China), Promega (Madison, USA), Beyotime (Shanghai, China) and Sigma-Aldrich (St. Louis, Missouri, USA), respectively. Antibodies against active caspase-3, active caspase-9, Akt, phospho-Akt (Ser473), BAX, Bcl-2, CDK4, CDK6, Cyclin D1, Cytochrome C, PARP were purchased from Abcam (Cambridge, UK). Antibodies to detect the protein levels of ERK1/2, phospho-ERK1/2 (Thy202/Tyr204), MEK1/2, phospho-MEK1/2 (Ser217/221) and phospho-PDK1 (Ser241) were obtained from Cell Signaling Technology (Boston, USA). PD0325901 and BX517 were obtained from MedChemExpress (Shanghai, China).

### MTT assay

Cell viability measurement was performed using the MTT assay. The appropriate number of cells was seeded into 96-well plates with 6 replicates and was treated as indicated. Subsequently, the cells were treated with 100 µL of MTT (5 mg/mL) at 37  °C for 4 h. After removal of the MTT solution, DMSO was added to the 96-well plates (150 µL/well), which were shaken. Optical density values were obtained at 490 nm using a microplate absorbance reader (Synergy HTX, BioTek, USA).

### Cell cycle detection

The Cell Cycle Detection Kit (KeyGEN, Nanjing, China) was employed to measure the cell cycle distribution. The cells were incubated with 9za at 0, 5, 10 µM for 24 h. Thereafter, the cells were detached by trypsin solution, washed with PBS twice and centrifuged at 1,000 rpm for 3 min. The collected cells were fixed with 70% cold ethanol at 4 °C overnight and then were incubated with 500 µL of PI/RNase working solution (PI:RNase A = 9:1). Cell cycle detection for each concentration of 9za in A549 and H1299 cells was conducted with three technical/ biological replicates. The cell DNA content was measured by flow cytometry (Sysmex, North Rhine-Westphalia, Germany) and analyzed using FCS express version 6 software.

### Cell apoptosis detection

Cell apoptosis detection was conducted using the Annexin V-FITC/PI Apoptosis Kit (MultiSciences, Hangzhou, China). After cell treatment as indicated, the cells were trypsinized, centrifuged and resuspended in binding buffer supplemented with Annexin V-FITC and PI. Following incubation, the cells were subjected to flow cytometry (Sysmex, North Rhine-Westphalia, Germany) and analyzed using FCS express version 6 software. In accordance with the manufacturer’s operating steps, the colorimetric TUNEL System (Promega, Madison, USA) was also employed to analyze the proportion of apoptotic cells.

### Mitochondrial membrane potential (MMP) assay

The Mitochondrial Membrane Potential Assay Kit with JC-1 (Beyotime, Shanghai, China) was adopted to detect the mitochondrial membrane potential (MMP). Using carbonyl cyanide 3-chlorophenylhydrazone (CCCP)-treated cells as positive controls, the DMSO- or 9za-treated cells were added to JC-1/culture media (1:1, v/v) and incubated at 37 °C for 20 min. Next, the cells were washed twice with JC-1 washing buffer. Subsequently, MMP was detected using a fluorescence microscope (Olympus, Tokyo, Japan). Red fluorescence indicates a high MMP of normal cell mitochondria, whereas green fluorescence indicates a low MMP of apoptotic cell mitochondria.

### Western blot detection

Cell lysates were obtained following lysis in RIPA buffer supplemented with PMSF. The extraction of mitochondrial and mitochondria-free protein was conducted using the Cell mitochondria Isolation Kit (Beyotime, Shanghai, China). Quantification of the extracted protein was completed using the BCA Protein Assay Kit (Thermo, Rockford, USA). Equal amounts of protein were electrophoresed on 8% or 10% SDS-PAGE gels and were transferred to PVDF membranes. Following blockade with 5% milk/TBST, the membranes were incubated with the primary antibodies and then the secondary antibodies. Visualization was achieved using enhanced chemiluminescence reagents (Merck Millipore, Billerica, MA).

### Statistical analysis

All the data were analyzed using SPSS 20.0 software. Significant differences between the groups were assessed by one-way ANOVA and independent-sample T test for multiple comparisons and single comparisons, respectively. The significance was stipulated as *P < 0.05, **P < 0.01 or ***P <0.001 compared with the controls.

## Results

### The dual MEK/PDK1 inhibitor 9za has potent cytotoxicity in NSCLC cells

To investigate the effect of 9za on NSCLC cell viability, A549 and H1299, two typical NSCLC cell lines, were treated with 0, 5, 10, 20, 40, 80, 160 µM of 9za for 12, 24 and 48 h, respectively, followed by the MTT assay. Additionally, to examine whether 9za has selective cytotoxicity in lung cancer cells, MRC-5 cells, a normal human lung fibroblast line, were also treated as above. As shown in Fig. 2B, 9za attenuated the cell viability of A549 and H1299 cells dose- and time-dependently. However, it exhibited little cytotoxic effect on MRC-5 cells compared with that on the two NSCLC cells. The IC_50_ values of 9za in the three cell lines for 12, 24 and 48 h are shown in Table 1.

**Table 1:** Median inhibitory concentration (IC_50_) of 9za on the viability of A549, H1299 and MRC-5 cells.

Cell lines	IC_50_ (µM)		
	12 h	24 h	48 h
A549	21.73 ± 2.35	10.86 ± 1.68	5.99 ± 1.38
H1299	27.23 ± 1.64	16.39 ± 1.11	7.59 ± 0.58
MRC-5	91.08 ± 1.12	68.66 ± 1.91	45.09 ± 1.55

**Notes.**

Data are indicated as the mean ± SEM.

IC_50_ concentration required for 50% inhibition

### Low concentrations of 9za retards the cell cycle in NSCLC cells

**Figure 3:**
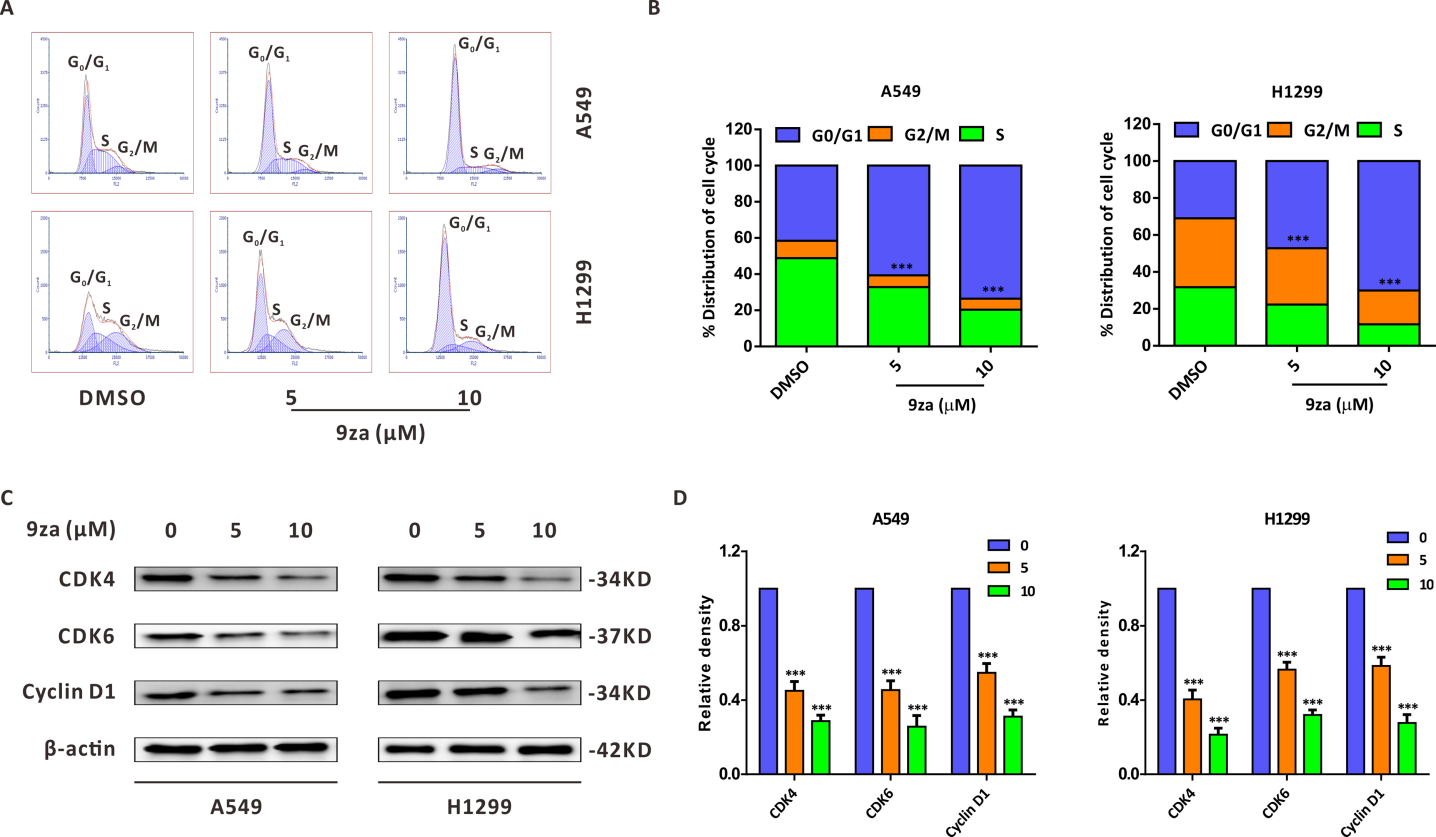
9za retards the cell cycle at G_0_/G_1_ phase at low concentrations in NSCLC cells. (A) The Cell Cycle Detection Kit and flow cytometry were employed to measure the cell cycle distribution in A549 and H1299 cells following 9za treatment at 0, 5, 10 µM for 24h. The black and red curves indicated cell distribution curve and cell fitted curve, respectively. The peaks of cell cycle phases were determined by the red fitted curves. (B) Statistical charts of the cell DNA content for (A) and the difference between the groups was analyzed according to the numbers of cells in G_0_/G_1_ phase (*n* = 3). (C) Cells were treated with 9za at 0, 5, 10 µM for 24h, and the extracted protein was subjected to Western blotting to detect the levels of CDK4, CDK6 and Cyclin D1. (D) Statistical graphs of the levels of CDK4, CDK6 and Cyclin D1 for (C) (*n* = 3). *P < 0.05, **P < 0.01 or ***P < 0.001 compared with the controls.

To explore whether 9za could affect cell cycle progression in NSCLC cells, the Cell Cycle Detection Kit and flow cytometry were applied to measure the cell cycle distribution. Figs. 3A–3B showed that 9za sharply raised the cell population at G_0_/G_1_ phase after treatment for 24 h at low concentrations (5 and 10 µM), accompanied by reduced cell numbers at S and G_2_/M phases. The cell cycle arrest effect of 9za was further confirmed by Western blot analysis. CDK4, CDK6 and Cyclin D1 are the major G_1_/S cell cycle checkpoint proteins which govern whether eukaryotic cells can enter S-phase from G_1_-phase *(VanArsdale et al., 2015)*. As indicated in Figs. 3C–3D, the cell cycle-related proteins CDK4, CDK6 and Cyclin D1 were decreased after 9za treatment. In summary, the data revealed that low concentrations of 9za could retard the cell cycle at G_0_/G_1_ phase.

### High concentrations of 9za promotes apoptosis in NSCLC cells

**Figure 4:**
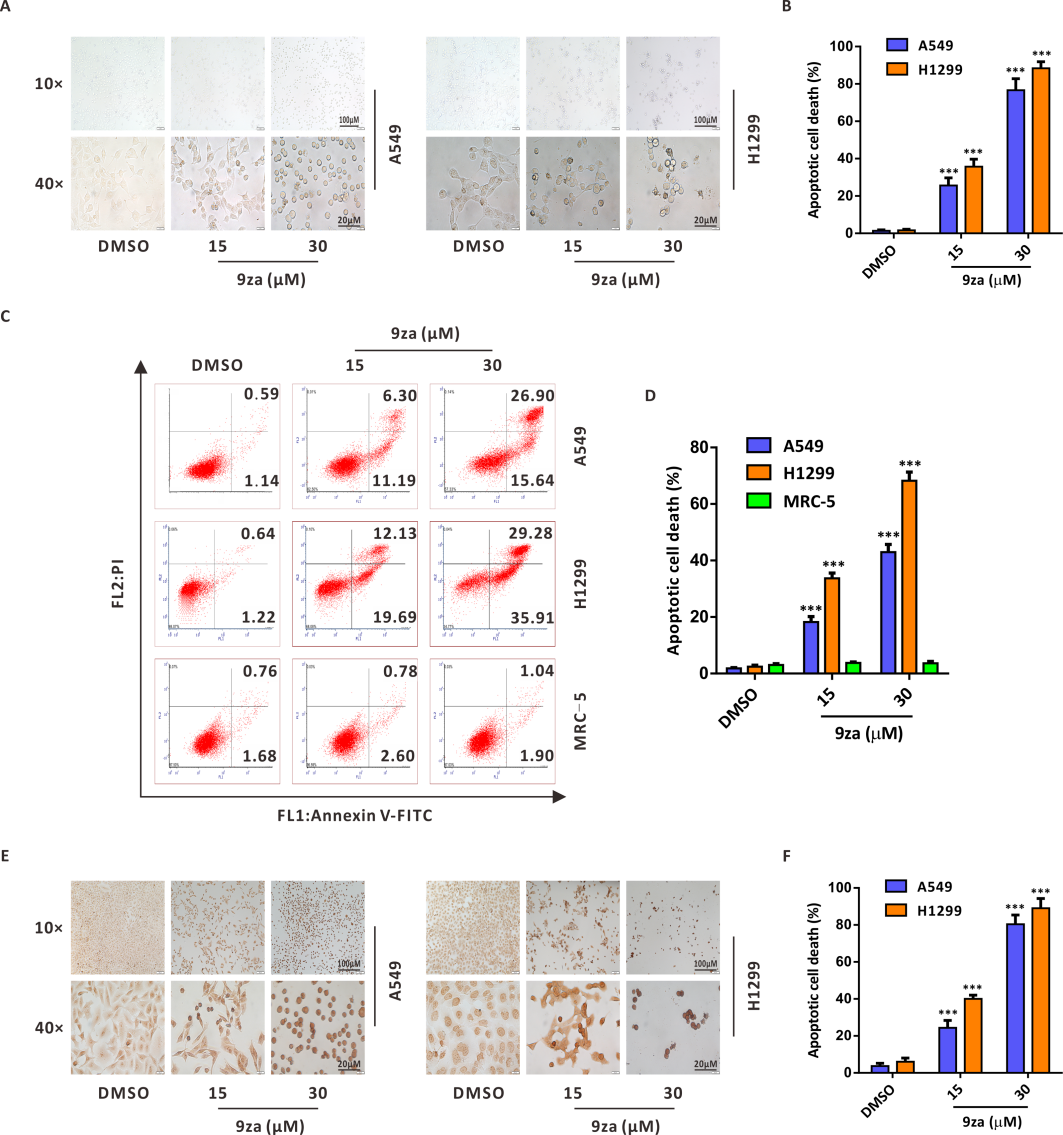
9za induces apoptosis at high concentrations in NSCLC cells. (A) Representative images of three view fields under a light microscope that were examined per technical replicate. Cells were exposed to 9za at 0, 15, 30 µM for 24 h and observed for cell morphology compared with the controls. Original magnification: upper, 10×; lower, 40×. (B) Statistical charts of the apoptotic cell death percentage for the upper part of (A) (*n* = 3). (C) Cells were treated with 9za at 0, 15, 30 µM for 24 h and measured using the Annexin V-FITC/PI Apoptosis Kit and flow cytometry. (D) Statistical histograms were calculated by flow cytometry analysis of (C) (*n* = 3). (E) Representative images of three view fields under a light microscope that were examined per technical replicate. Cells were treated with 9za at 0, 15, 30 µM for 24 h and stained with the TUNEL system. The TUNEL-positive cells (i.e., apoptotic cells) were visualized compared with the controls. Original magnification: upper, 10×; lower, 40×. (F) Statistical graphs of the apoptotic cell death percentage for the upper part of (E) (*n* = 3). *P < 0.05, **P < 0.01 or ***P < 0.001 compared with the controls.

To investigate whether 9za might induce cell apoptosis in NSCLC and MRC-5 cells, we firstly examined the number and morphology features of 9za-treated cells under a light microscope in NSCLC cells. As exhibited in Figs. 4A–4B, morphology observation showed that 9za-treated cells became rounded and shed when compared to the control cells, which hinted that 9za might induce apoptosis in NSCLC cells. Then the Annexin V-FITC/PI Apoptosis Kit, a classical cell apoptosis detection method, was employed to examine cell apoptosis after 9za treatment. The results indicated that, at high concentrations (15 and 30 µM), 9za obviously elevated the apoptotic cell population, including showing apoptosis in the early stage (Annexin V +/PI -) and in late stage (Annexin V +/PI +) compared with the controls in NSCLC cells, but had no proapoptotic effect in MRC-5 cells (Figs. 4C–4D). Similar results were obtained from the colorimetric TUNEL assay which demonstrated that 9za could significantly increase the cell percentage of TUNEL-positive cells (Figs. 4E–4F). The above data revealed that high concentrations of 9za might induce cell apoptosis in NSCLC cells.

### 9za induces mitochondria-mediated apoptosis in NSCLC cells

**Figure 5:**
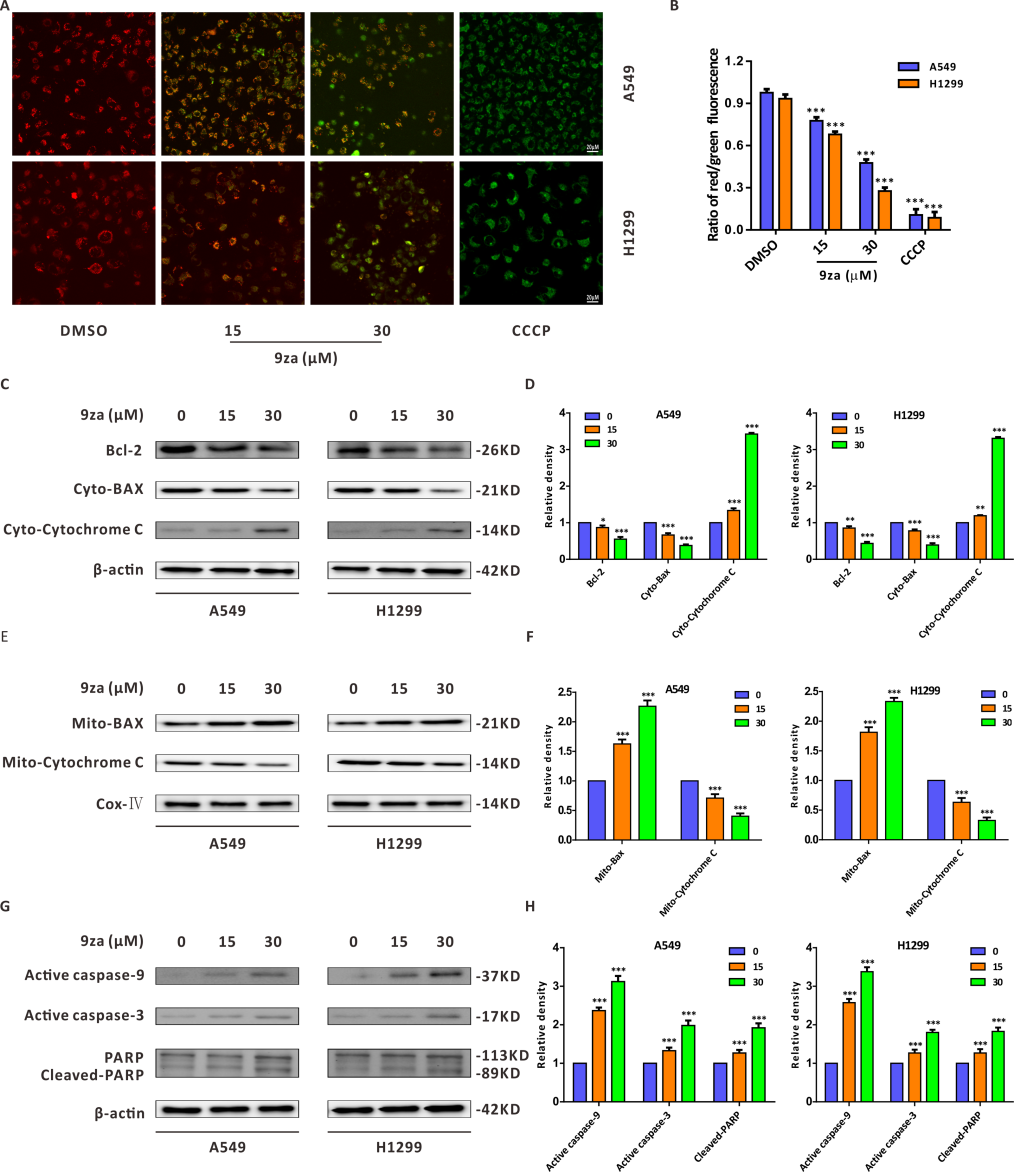
9za induces mitochondria-mediated apoptosis in NSCLC cells. (A) Representative images of three view fields under a confocal fluorescence microscope that were examined per technical replicate. MMP was detected by JC-1 staining after the cells were treated with 9za at 0, 15, 30 µM for 24 h. Original magnification: 40×. (B) Statistical histograms of the ratios of red/green fluorescence for (A) (*n* = 3). (C and E) Cells were handled with 9za at 0, 15, 30 µM for 24 h. If necessary, the cytoplasm and mitochondria were separated using the Cell Mitochondria Isolation Kit. The levels of Bcl-2, BAX in the cytoplasm, Cytochrome C in the cytoplasm, BAX in the mitochondria and Cytochrome C in the mitochondria were analyzed by immunoblotting. (D and F) Statistical graphs of the levels of Bcl-2, BAX in the cytoplasm, Cytochrome C in the cytoplasm, Bcl-2 in the mitochondria and Cytochrome C in the mitochondria quantified through Western blotting analysis of (C) and (E), respectively (*n* = 3). (G) Immunoblotting was adopted to measure the levels of active caspase-9, active caspase-3 and cleaved-PARP after the cells were treated with 9za at 0, 15, 30 µM for 24 h. (H) Statistical diagrams of the relative levels of active caspase-9, active caspase-3 and cleaved-PARP (*n* = 3). *P < 0.05, **P < 0.01 or ***P < 0.001 compared with the controls.

There are two main apoptosis types including the extrinsic apoptosis, i.e., death receptor pathway, and the intrinsic apoptosis, i.e., mitochondrial pathway (Fan et al., 2016; Huang et al., 2015). It was already confirmed that the downregulation of the mitochondrial membrane potential (MMP) is the first step in mitochondrial apoptosis, which results in the permeabilization of the mitochondrial membrane and release of Cytochrome C from the mitochondria into the cytoplasm (Chen et al., 2018; Li et al., 2014). To identify whether 9za-mediated cell apoptosis was initiated by the mitochondrial pathway in NSCLC cells, JC-1 staining assay was adopted to detect MMP (Xie et al., 2018). As shown in Figs. 5A–5B we regarded DMSO-treated cells and carbonyl cyanide 3-chlorophenylhydrazone (CCCP)-treated cells as the negative controls and positive controls, respectively. The negative controls displayed red fluorescence, whereas the positive controls exhibited almost green fluorescence. The ratios of red/green fluorescence were significantly decreased after 9za exposure in NSCLC cells. These data showed that 9za could downregulate MMP and propel the depolarization of the mitochondrial membrane. To further elucidate the proapoptotic mechanism of 9za, we investigated the expression of mitochondrial apoptosis-associated proteins by Western blotting. As presented in Figs. 5C–5F, immunoblotting showed that the protein level of Bcl-2 was obviously reduced following 9za incubation. Additionally, the expression levels of BAX in the mitochondria and Cytochrome C in the cytoplasm were increased. By contrast, the protein levels of BAX in the cytoplasm and Cytochrome C in the mitochondria were downregulated. Moreover, Western blot analysis revealed that the expression levels of active caspase-9, active caspase-3 and cleaved-PARP were clearly enhanced after 9za incubation in NSCLC cells (Figs. 5G–5H). Together, these results indicated that, depending on the caspase cascade, 9za induced mitochondrion-mediated apoptosis in NSCLC cells.

### 9za induces cell apoptosis through the MEK/ERK and PDK1/Akt signaling pathways in NSCLC cells

**Figure 6:**
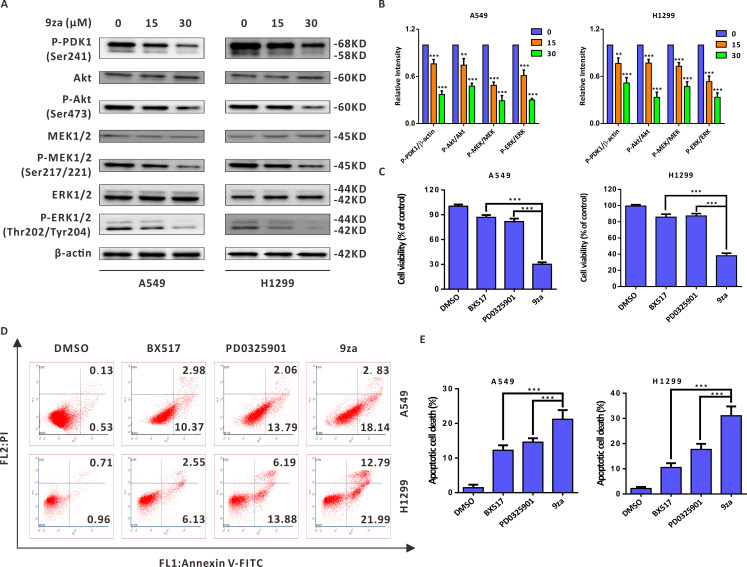
9za induces cell apoptosis through the MEK/ERK and PDK1/Akt signaling pathways in NSCLC cells. (A) The phosphorylation levels of PDK1 (Ser241), Akt (Ser473), MEK1/2 (Ser217/221) and ERK1/2 (Thr202/Tyr204) were analyzed by Western blotting following cell exposure to 9za at 0, 15, 30 µM for 24h. (B) Statistical diagrams of the relative intensity of P-PDK1/β-actin, P-Akt/Akt, P-MEK/MEK and P-ERK/ERK (*n* = 3). (C) The cells were exposed to DMSO, BX517, PD0325901 and 9za at the same concentration (20 µM) for 24 h. Cell viability was measured by the MTT assay (*n* = 6). (D) The Annexin V-FITC/PI Apoptosis Kit was applied to investigate cell apoptosis after the cells were treated with DMSO, BX517, PD0325901 and 9za at the same concentration (20 µM) for 24 h. (E) Bar graphs were calculated by flow cytometry analysis for (D) (*n* = 3). *P < 0.05, **P < 0.01 or ***P < 0.001 compared with the controls.

Given that 9za was designed and synthesized as a dual MEK/PDK1 inhibitor, it is necessary to examine whether 9za could suppress the MEK and PDK1 signaling pathways. Western blotting results indicated that 9za reduced the phosphorylation levels of PDK1, Akt, MEK1/2 and ERK1/2 (Figs. 6A–6B), illuminating that 9za simultaneously inhibited the MEK/ERK and PDK1/Akt signaling pathways. To compare the anti-tumor activity of the single MEK or PDK1 inhibitor and the dual MEK/PDK1 inhibitor in NSCLC cells, the same concentration (20 µM) of BX517 (a PDK1 inhibitor), PD0325901 (a MEK inhibitor) and 9za (a dual MEK/PDK1 inhibitor) were used to clarify their potential effects on NSCLC cell viability and cell apoptosis. The MTT assay (Fig. 6C) showed that 9za enhanced the efficacy of cell proliferation inhibition compared with BX517 or PD0325901 treatment alone. A similar conclusion was obtained from Annexin V-FITC/PI apoptosis detection (Figs. 6D–6E). The above data illuminated that 9za was indeed a dual MEK/PDK1 inhibitor, and the dual suppression of the MEK/ERK and PDK1/Akt signaling axes strengthened the cytotoxicity and cell apoptosis compared with the single inhibition of the MEK or PDK1 signaling pathway in NSCLC cells.

## Discussion

An increasing number of studies has verified that the RAF/MEK/ERK and PI3K/PDK1/Akt signaling axes are hyperactivated in various tumors, making them potential targets for new anti-cancer agents (Hrustanovic et al., 2015; McCarroll et al., 2015). Numerous structurally diverse small-molecule compounds, such as MEK inhibitors (e.g., PD0325901 and Selumetinib) and PI3K inhibitors (e.g., Buparlisib and Idelalisib), have been discovered by targeting the RAF/MEK/ERK or PI3K/Akt/mTOR signaling pathways in the search for potential drugs for human cancers (Houédé & Pourquier, 2015; Yu et al., 2015). However, the drug resistance was soon acquired due to the crosstalk and existence of a compensation mechanism between the two axes. There is growing evidence that the simultaneous blockade of both axes might be remarkably more effective than the blockade of either pathway alone (Ciuffreda et al., 2014). Although the combined treatment of the inhibitors against PI3K/PDK1/AKT and RAF/MEK/ERK signaling axes might reasonably postpone the occurrence of drug tolerance, prescription components and drug interactions hamper the clinical promotion of the combinational therapy (Meng et al., 2010; Temraz, Mukherji & Shamseddine, 2015). Presently, the discovery of dual inhibitors simultaneously targeting the PI3K/PDK1/Akt and RAF/MEK/ERK signaling pathways has become a research hotspot in the pursuit of targeted anti-tumor medicine. Although some dual inhibitors targeting these two pathways have been reported in recent years, there is still a lack of diversity in the chemical scaffolds to achieve synergistic blockade against the two signaling pathways, which might be expected to reduce the drug resistance(Li et al., 2010; Park, Lee & Hong, 2012).

Based on the above reasons, considering PD0325901 (a MEK inhibitor) and BX517 (a PDK1 inhibitor) as the lead compounds, we exploited a new type of dual MEK/PDK1 inhibitor by merging the active structural skeletons (Yu et al., 2019). The novel type of dual inhibitors includes a diphenylamine main frame of PD0325901, which was designated to preserve the MEK hydrophobicity, and an indolone core skeleton, which was appointed to maintain the suppressive activity against PDK1. Our previous work confirmed that 9za can induce autophagy through PDK1/Akt/mTOR signaling pathway (Liu et al., 2019b). This work further verified that 9za is indeed a dual inhibitor against MEK/ERK and PDK1/Akt signaling axes. Furthermore, 9za treatment strengthened the effect of cytotoxicity and pro-apoptosis compared with BX517 or PD0325901 treatment alone, which exactly met our expectation and agreed with the previous literature. Although we have only investigated the anti-lung cancer activity of 9za *in vitro* until now, we can anticipate the potent anti-tumor effect *in vivo* based on that 9za is a dual MEK/PDK1 inhibitor and has stronger anti-cancer efficacy than the single MEK/PDK1 inhibitor. Due to the advantage of low drug resistance caused by this new type of dual MEK/PDK1 inhibitor, as has been suggested in the literature, further investigation might focus on exploring the drug resistance of 9za.

Programmed cell death includes apoptosis, necroptosis, pyroptosis, ferroptosis and others, among which, apoptosis is a metabolically active process of cell death occurring with characteristic morphologic features: cell membrane shrinkage, plasma membrane blebbing, nuclear chromatin condensation and nuclear fragmentation (Galluzzi et al., 2018). In this study, the morphology observation clearly showed that 9za-treated cells became rounded and shed, which hinted that 9za might promote cell apoptosis in NSCLC cells. Additionally, both the results of Annexin V-FITC/PI apoptosis detection and the colorimetric TUNEL assay demonstrated that 9za treatment significantly induced cell apoptosis. Apoptosis is mediated by two major signaling cascades: the intrinsic and extrinsic apoptosis pathways (Tait & Green, 2010; Taylor, Cullen & Martin, 2008). Intrinsic apoptosis generally occurs because of a disruption of cellular homeostasis, while extrinsic apoptosis occurs because of extracellular signaling by death receptors. Mitochondrial outer membrane permeabilization causes cells to undergo intrinsic apoptosis. Although intrinsic and extrinsic apoptosis have unique initiating steps, both converge to the cascade activation of a set of caspases. Initiator caspases (such as caspase 8 or 9) begin a nest of proteolytic steps to activate executioner caspases (for example caspase 3 or 7). In this study, JC-1 staining results revealed that 9za treatment could promote mitochondrial membrane depolarization and downregulate the mitochondrial membrane potential (MMP) at high concentrations. Immunoblotting results showed that 9za reduced the Bcl-2 level, simultaneously upregulated the levels of BAX in the mitochondria and Cytochrome C in the cytoplasm and downregulated the levels of BAX in the cytoplasm and Cytochrome C in the mitochondria. Moreover, the expression levels of active caspase-9, active caspase-3 and cleaved-PARP were markedly enhanced following 9za treatment in NSCLC cells. All of the above data suggested that 9za induces mitochondria-mediated cell apoptosis in a caspase-dependent manner at high concentrations (>10 µM) in NSCLC cells.

Another pivotal mechanism that has contributed to the anti-tumor activity of chemotherapeutic drugs is cell cycle arrest, which accumulates cancer cells at a specific phase and halts the progress of the cell cycle (Lin et al., 2018). Our results showed that 9za could retard the cell population at G_0_/G_1_ phase at low concentrations (<10 µM). Additionally, cell cycle arrest after 9za treatment could be explained by the decreased levels of CDK4, CDK6 and Cyclin D1, which are consistent with the previous literature (Liu et al., 2019a; Zhang et al., 2018). It is notable that when the concentration was less than or equal to 10 µM, 9za showed a trend of dose-dependent cell cycle arrest in G_0_/G_1_ phase. However, cell apoptosis was obvious and the tendency of cell cycle arrest was disrupted when the concentration of 9za exceeded 10 µM, which was the reason why the specific low (5/10 uM) and high (15/30 uM) concentrations of 9za were used respectively to detect cell cycle and cell apoptosis in the study.

**Figure 7:**
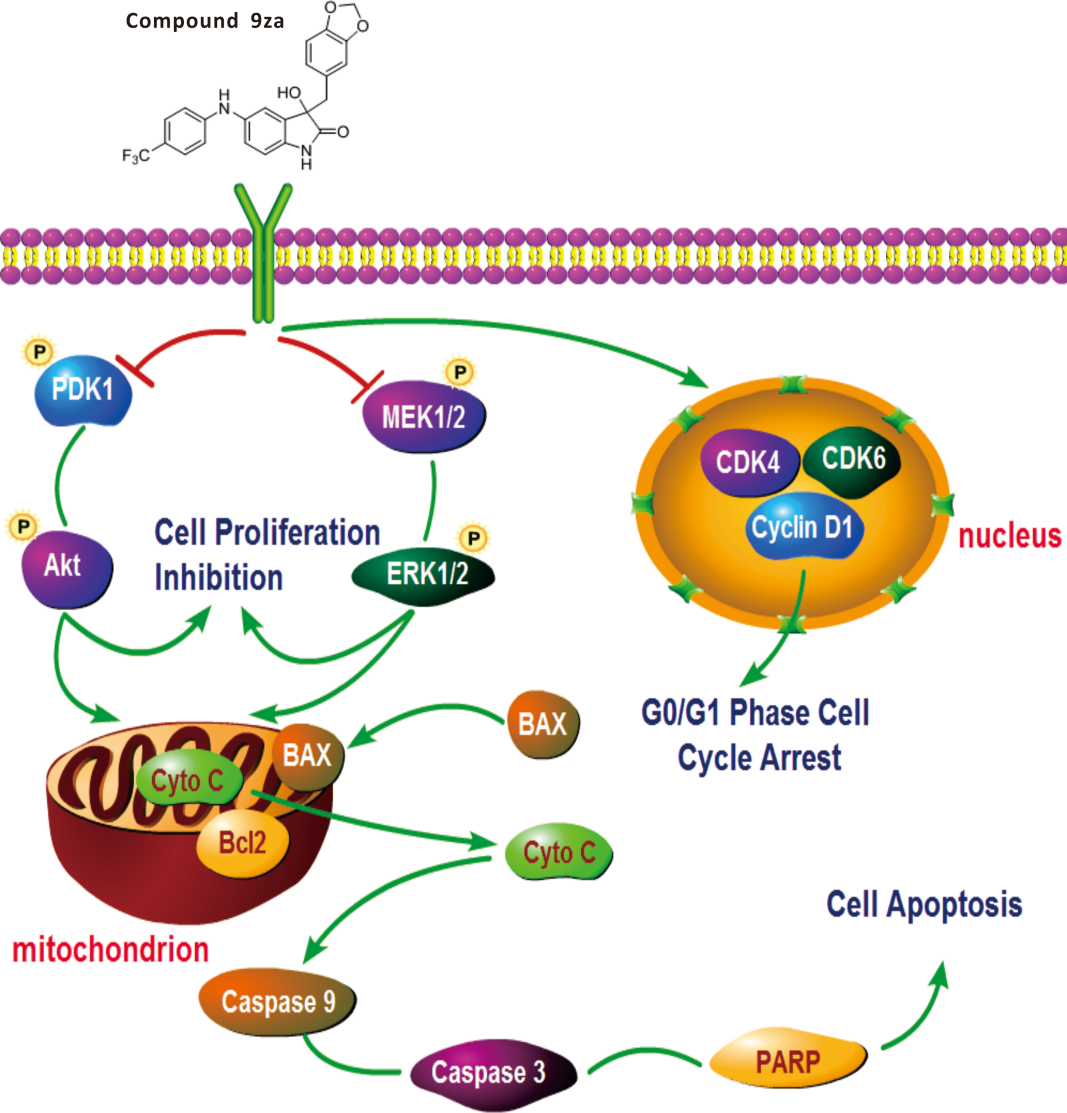
General schematic of the effect of 9za on cell cycle arrest, cell proliferation inhibition and mitochondrial apoptosis in NSCLC cells. After treatment, 9za suppresses the dual MEK/ERK and PDK1/Akt signaling pathways, leading to cell proliferation inhibition and mitochondrial apoptosis via the release of Cytochrome C from mitochondria into the cytoplasm and activation of caspase-9, caspase-3 and cleavage of PARP. Moreover, 9za suppressed the activities of CDK1, CDK6 and Cyclin D1, resulting in cell cycle arrest at G_0_/G_1_ phase.

## Conclusion

This study verified that 9za could block the dual MEK/ERK and PDK1/Akt signaling pathways. 9za at low concentrations down-regulated the protein expression levels of CDK4, CDK6 and Cyclin D1, resulting in cell cycle arrest at G_0_/G_1_ phase. The high concentrations of 9za caused the release of Cytochrome C from the mitochondria into the cytoplasm and BAX to the mitochondria from the cytoplasm, and promoted caspase-9, caspase-3 activation and PARP cleavage, which was consistent with the characteristics of mitochondrial apoptosis (Fig. 7). In short, this study confirmed that 9za had cytotoxic and pro-apoptotic effects in NSCLC cells and provided an experimental foundation to develop 9za as a novel drug candidate against lung carcinoma.

##  Supplemental Information

10.7717/peerj.9981/supp-1 File S1 Full-length uncropped blots of Fig. 3

10.7717/peerj.9981/supp-2 File S2 Full-length uncropped blots of Fig. 5

10.7717/peerj.9981/supp-3 File S3 Full-length uncropped blots of Fig. 6

## Additional Information and Declarations

### Data Availability

The following information was supplied regarding data availability: The raw measurements are available as Supplementary Files.
