# Supplementary figures and images for: A novel dual MEK/PDK1 inhibitor 9za retards the cell cycle at G0/G1 phase and induces mitochondrial apoptosis in non-small cell lung cancer cells
Source: PeerJ. 2020 Oct 2;8:e9981. doi: 10.7717/peerj.9981 (PMC7537639; doi:10.7717/peerj.9981)

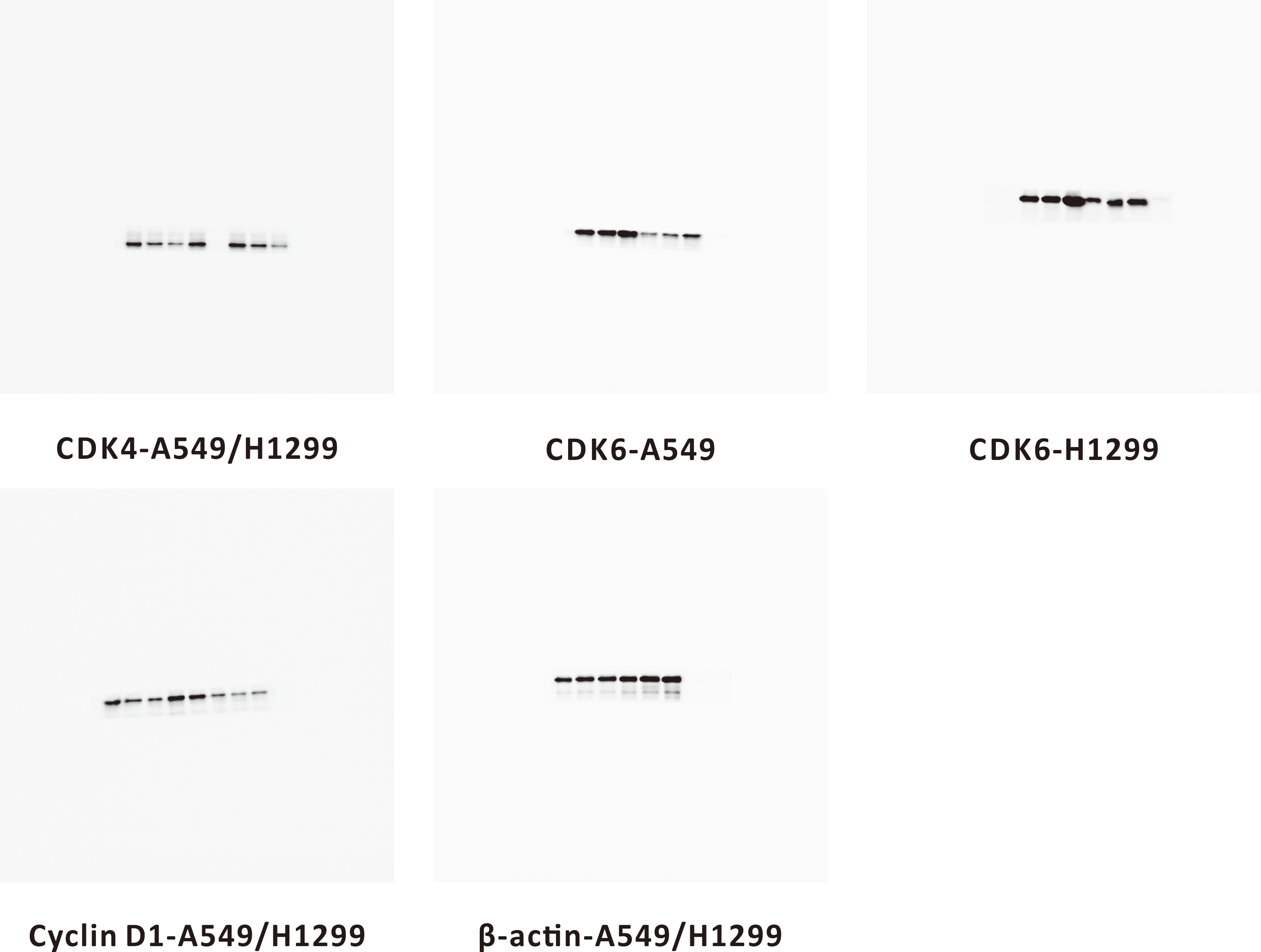

Supplement: File S1 [file peerj-08-9981-s001.png]
